# NanoTouch: intracellular recording using transmembrane conductive nanoparticles

**DOI:** 10.1152/jn.00359.2019

**Published:** 2019-09-04

**Authors:** Mitsuyoshi L. Saito

**Affiliations:** Ion Chat Research Corporation, Saitama, Japan

**Keywords:** conductive nanoparticles, intracellular recordings, needleless electrophysiology

## Abstract

Observations of the electrophysiological properties of cells are important for understanding cellular functions and their underlying mechanisms. Short action potentials in axons are essential to rapidly deliver signals from the neuronal cell body to the terminals, whereas longer action potentials are required for sufficient calcium influx for transmitter release at the synaptic terminals and for cardiomyocyte and smooth muscle contractions. To accurately observe the shape and timing of depolarizations, it is essential to measure changes in the intracellular membrane potential. The ability to record action potentials and intracellular membrane potentials from mammalian cells and neurons was made possible by Ling and Gerard’s discovery in 1949, when they introduced sharp glass electrode with a submicron sized tip. Because of the small tip size, the sharp glass electrode could penetrate the cell membrane with little damage, which was one of the major breakthroughs in cellular electrophysiology and is the basic principle of the intracellular recording technique to date, providing the basis for further innovation of patch-clamp electrophysiology. I report a proof-of-principle demonstration of a novel method for recording intracellular potentials without penetrating the cell membrane using glass electrodes. We discovered that magnetically held transmembrane conductive nanoparticles can function as an intracellular electrode to detect transmembrane membrane potentials similar to those obtained by the conventional patch-clamp recording method.

**NEW & NOTEWORTHY** To accurately observe the shape of action potentials, it is essential to perform intracellular recordings. I present a method to record intracellular potentials using magnetically held magnetic conductive nanoparticles in the membrane as an electrode. These nanoparticles function similarly to a conventional intracellular microelectrode. This is the first report to apply conductive nanoparticles to detect action potentials in the form of electrical signals.

## INTRODUCTION

Bioactivities in the brain and heart are studied by measuring complex electrical signals in the form of extracellular compound action potentials using electroencephalography and electrocardiography, respectively. However, to investigate these transmembrane electrical activities in detail, it is necessary to place a voltage-sensing probe inside the cell. This is commonly achieved by inserting a high-resistance glass microelectrode into the cell or by rupturing a patch of the membrane under a tightly sealed patch electrode, in which the opening of the patch pipette senses the intracellular membrane potential (Bretag 2017; Hille 2001; Junge 1981).

The introduction of the sharp glass electrode technique by Ling and Gerard (1949) is one of the major breakthroughs in cellular electrophysiology. Because of the submicrometer size of the small tip, the sharp glass electrode has the ability to penetrate the cell membrane with little damage allowing for the accurate measurement of the intracellular potential. The sharp electrode method is adapted for almost all conceivable cell types from unicellular organisms to neurons in the living brain (Verkhratsky and Parpura 2014) and is the basic principle of the intracellular recording technique to date.

**Fig. 1.**
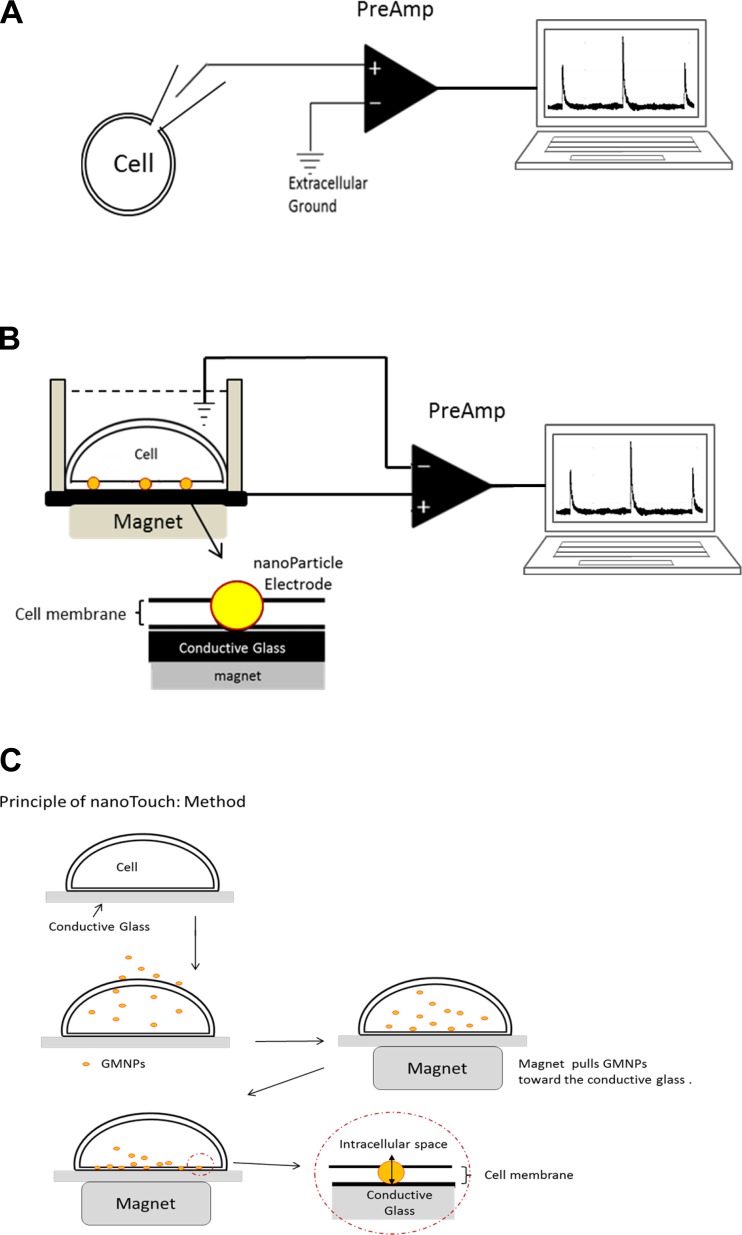
Conformation of a conventional intracellular recording method and the concept of NanoTouch: gold-coated magnetic nanoparticles (GMNPs) are integral to the intracellular recording method. *A*: conformation of conventional intracellular recording. A glass microelectrode is inserted into the cell. Outputs from the electrode and the reference electrode placed in the bath are fed to the amplifier (PreAmp), which allows detection of membrane potential changes. *B*: the concept of “NanoTouch”: a GMNP-based intracellular recording method. An intracellular recording configuration using a nanoparticle intracellular voltage sensor was assembled and comprised *1*) polyethylene glycol-stabilized GMNPs (50 nm in diameter), *2*) a neodymium magnet (Moritoku Corp.), and *3*) a conductive glass slide (CG; 2.5 cm × 2.5 cm, fluorine-doped tin oxide; Kenis) that functions as a cell plating substrate and as a connection material to connect GMNPs with the amplifier when GMNPs penetrate the bottom cell membrane and make contact with (touch) the CG. *C*: cells were plated on a CG slide and GMNPs were delivered to the cytosol using polyethyleneimine (PEI; dendrimers). Activated dendrimer (SuperFect, Qiagen) or pore-forming peptides (streptolysin O, SLO) can be used in place of PEI (Lévy et al. 2010). Prior to this step, it is important to confirm that the plated cells have formed a sheet, because any gaps in the cell sheet will cause shunt circuits between the bath medium and the CG surface. Next, a magnet was placed below the CG recording chamber, which pulled GMNPs toward the bottom of the cells, which had adhered to the CG. Finally, as GMNPs penetrated and spanned the membrane, the electrical connection between the intracellular compartment of the cell and the CG substrate was monitored with a conventional patch-clamp amplifier. With the reference electrode set in the bath solution and the CG connected to the amplifier inputs, the GMNPs functioned as an intracellular electrode by bridging the cellular membrane and connecting the cytosolic compartment to the CG surface (*B*).

Another technical breakthrough came when Neher and Sakmann (1976) developed the patch-clamp technique that detects ionic current movements through single ion channels, which are the building blocks that shape action potentials. The patch-clamp technique was further refined by Sigworth and Neher (1980) with the introduction of the “gigaseal recording.” The patch-clamp method is now widely adapted as a core technique for electrophysiological studies (Fig. 1*A*) and is regarded as the gold standard for cellular electrophysiology research.

We discovered that gold-coated magnetic conductive nanoparticles, whose diameters were larger than the thickness of the cell membrane, could function as an intracellular electrode when they are held in the membrane. In this report, I present a proof-of-principle demonstration of how magnetically held transmembrane conductive nanoparticles can function as an intracellular voltage sensor to detect transmembrane membrane potentials in cultured cells.

## METHODS

### Solutions

Composition of the extracellular and intracellular solutions was as follows: extracellular solution contained (in mM) 126 NaCl, 4 KCl, 1.8 CaCl_2_, 1 MgCl_2_, 24 HEPES, and 10 glucose (pH 7.4 adjusted with NaOH), and intracellular solution contained (in mM) 130 KCl, 5 MgCl_2_, 5 EGTA, 4 Tris-ATP, and 10 HEPES (pH 7.2 adjusted with KOH). Intracellular solution was for the patch-clamp experiments only. All chemicals were purchased from Sigma-Aldrich (St. Louis, MO).

### Cell Culture Conditions

Chinese hamster ovary (CHO) culture medium contained minimum essential medium Eagle (Sigma-Aldrich), 10% FBS (BioWest), and 5% 40 mM l-glutamine (Wako Pure Chemical Industries). Human embryonic kidney (HEK-293) culture medium contained DMEM (Sigma-Aldrich) and 10% FBS (BioWest).

### Current- and Voltage-Clamp Experiments

Current-clamp experiments were carried out using an Axopatch 200A (Axon Instruments, Union City, CA). Data were digitized using a Digidata 1332a (Axon Instruments). Data capture and voltage control were performed using Clampex (pClamp9.0; Axon Instruments). Data were analyzed with Clampfit10.6.2.2 (Molecular Devices, San Jose, CA).

For analyses, mean, SD, SE, and *t* test calculations were carried out using Excel (Microsoft, Redmond, WA). All calculated data are means ± SE. Plots were constructed using Excel (Microsoft) and SigmaPlot 9 (Systat Software, San Jose, CA).

Capacitance and input resistance were calculated by fitting the discharging phase of voltage traces using the following double-exponential decay equation:

$$y=a\cdot exp\left(-t/\tau -a\right)+m\cdot exp\left(-t/\tau -m\right),$$

where *y* is membrane potential (*V*_m_), *a* and *m* are amplitudes, τ − *a* and τ − *m* are decaying time constants, *t* is time, and τ = *RC*, where *R* is membrane resistance and *C* is cell capacitance.

Double-exponential fit was necessary because discharging voltage traces consist of the access resistance (*R*_acc_, number of transmembrane conductive nanoparticles) and the membrane input resistance (*R*_m_). Contributions of *R*_acc_ and *R*_m_ were determined as *a* and *m*, and contributions of decay time constant were τ − *a* and τ − *m*, respectively.

### Channelrhodopsin Cloning and Cell Line Development

The channelrhodopsin mutational variant hChRWR was PCR amplified from cDNA (1,073 bp; Wang et al. 2009) using Phusion DNA polymerase (New England Biolabs, Ipswich, MA) and subcloned into pD603 (carrying puromycin selection; DNA2.0, Menlo Park, CA) using InFusion (TakaraBio). The following primers were used: sense primer, accatggctcggagaccctggct; and antisense primer, cttgcctgtccctttgttga. CHO cells (JCRB Cell Bank) were transfected with the hChRWR-pD603 vector using SuperFect transfection reagent (Qiagen).

### Generation of a HEK Cell Line Stably Expressing Nav1.5/Kir2.1.

HEK-293 cells (JCRB Cell Bank) were transfected with Nav1.5-pcDNA3.1 (carrying hygromycin selection; Invitrogen) and Kir2.1-pD608 (carrying blasticidin selection; DNA2.0) using SuperFect transfection reagent (Qiagen). The Nav1.5 expression vector was constructed by subcloning *SCNA5A* (GenBank accession no. BC140813; SourceBioscience, UK) into pcDNA3.1(−). BC140813 *SCNA5A* cDNA contains two embryonic exons; therefore, these exons were replaced by a PCR method with corresponding exons from a human heart cDNA (Zyagen, San Diego, CA).

Kir2.1 (NCBI reference sequence NM_000891, *KCNJ2*, 1,284 bp) was cloned from cardiomyocyte cDNA using the nesting PCR method. The cDNA was generated from total RNA extracted from cardiomyocyte-like cells derived from induced pluripotent stem cells (Cellular Dynamics International, Madison WI).

The following primers were used in these PCR reactions. First-round PCR primers were Kir2.1 1st, sense, CCAAAGCAGAAGCACTGGAG; and Kir2.1 1st, antisense, CTTTGAAACCATTGTGCTTGCC. Second-round PCR primers were Kir2.1 2nd, sense, atgggcagtgtgcgaaccaac; and Kir2.1 2nd, antisense, tcatatctccgactctcgccg.

The Kir2.1 PCR product was subcloned into pD608 vector. The Kir2.1 sequence was confirmed by DNA sequencing. All oligos were purchased from Integrated DNA Technologies (Coralville, IA).

Electrophysiological validation of the HEK cell line that stably expressed Nav1.5 and Kir2.1 channels using conventional patch-clamp recordings under voltage clamp was carried out using the following voltage-clamp protocols.

#### Nav1.5 activation protocol.

The membrane potential was held at −80 mV. Prepulse (−120 mV, 500 ms) was followed by 200-ms depolarization steps from −80 to 40 mV in 10-mV steps.

#### Kir2.1 activation protocol.

The membrane potential was held at −80 mV. The membrane potential was first stepped to −20 mV (200 ms) and then from −40 to −120 mV (200 ms) in 10-mV steps.

### Delivery of Polyethylene Glycol-Stabilized Gold-Coated Magnetic Nanoparticles into Cells by Polyethyleneimine

Delivery of gold-coated magnetic nanoparticles (GMNPs; 50 nm in diameter) into cells was performed using a modified form of a previously described method (https://labs.fccc.edu/yen/docs/PEI%20preparation.pdf). The properties of COOH-polyethylene glycol (PEG) 3,000-Da stabilized GMNPs (50 nm) are summarized on the manufacturer’s web site (https://nanoimmunotech.eu/uploads/shop/productos/Datasheet%20NITmagold%20COOH-PEG%2050%20nm%2020190212%20%284%29.pdf). Polyethyleneimine (PEI; 1 mg; M_w_ 750,000; Sigma-Aldrich) was dissolved in 1 mL of double-distilled H_2_O (ddH_2_O) solution (pH adjusted to 7.0) and filtered (0.2-μm filter; Minisart, Sartorius, DE). The solution was active for 2 wk. The PEI solution was diluted 100-fold with ddH_2_O just before use.

CHO or HEK cells were harvested and counted, and 5 × 10^5^ cells were rinsed once with PBS(−). GMNP-PEI solution consisted of 5 μL of 100-fold-diluted PEI stock solution, 80 μL of PEG-stabilized GMNPs, 20 μL of 5× HEPES-buffered physiological saline (HBPS; in mM: 24 HEPES, 126 NaCl, 4 KCl, 1 CaCl_2_, 1 MgCl_2_, and 10 glucose). The mixture was incubated at room temperature for 15 min. hChRWR-CHO cells were washed once with PBS(−), and the GMNP-PEI mixture was applied. The culture was left for 15 min in a 37°C incubator before experiments.

PEG-stabilized GMNPs (50 nm in diameter) were purchased from NanoImmunotech (Spain), neodymium magnets were obtained from Moritoku (Japan), and fluorine-doped tin oxide (FTO) conductive glass slides were obtained from Kenis (Japan).

### Delivery of GMNPs into Cells by Streptolysin O

We modified the method reported by Walev et al. (2001) to introduce GMNPs to cells. First, streptolysin O (SLO; Wako Pure Chemical) was activated by reduction with DTT (Nacalai Tesque). Stock solutions of SLO with a final concentration of ~5 U/μL were prepared.

HEK cells (5 × 10^5^) were rinsed with PBS(−) and resuspended in 20 μL of PEG-GMNP solution (NanoImmunotech) and 5 μL of 5× HBPS (1 mM Ca^2+^, 1 mM Mg^2+^). These cells were incubated in a 37°C incubator for 12 min in the presence of 1 μL of SLO. The pore-forming activities of SLO were inactivated by adding DMEM containing 10% FBS (0.5–1 mL) for 20 min or longer.

## RESULTS

### 

The idea to use nanoparticles for electrophysiological recordings came from reports showing how silica nanoparticles can interact with a lipid bilayer membrane and alter its electrical properties (Carney et al. 2013; de Planque et al. 2011). We considered that if conductive nanoparticles could be held across a cell membrane, they could provide a minimally disruptive, low-resistance pathway across the membrane to allow the detection of changes in intracellular membrane potentials. A schematic depiction of this method is shown in Fig. 1*B*.

As with transmembrane proteins, when a conductive nanoparticle with a diameter that is larger than the thickness of the cell membrane is held in the membrane, both intracellular and extracellular regions of the nanoparticle will protrude from the lipid bilayer. Consequently, the region of the nanoparticle exposed to the cytosol can detect changes in intracellular membrane potential, raising the possibility that these changes can be measured from the extracellular part of the nanoparticle. By connecting the extracellular region of the conductive nanoparticles and the bath electrode to amplifier inputs, we successfully made intracellular current-clamp recordings from cultured cells similar to those obtained by conventional glass electrode recording methods.

To achieve a conductive nanoparticle-based intracellular recording method, we assembled the following components: *1*) PEG-stabilized GMNPs (50 nm in diameter), *2*) a neodymium magnet (180–220 mT), and *3*) a conductive glass slide (CG; fluorine-doped tin oxide glass), which functions as a cell plating substrate and a connection cable to connect GMNPs with an amplifier when GMNPs penetrate the cell membrane and make electrical contact with the CG. The CG surface was covered with a nonconductive plastic mask, which had a 2-mm-diameter opening in the center. Cells plated in this opening were subjected to recordings. We call this method “NanoTouch” because the transmembrane conductive nanoparticles must touch the extracellular conductive glass for successful recording.

In this method, intracellular voltage sensors, GMNPs, are magnetically held in the membrane, electrically bridging the cytosol with the extracellular CG substrate. The diameter of GMNPs used was 50 nm, which is large enough to span the 7.5- to 10-nm-thick cell membrane (Hine 1999). Cytosolic GMNPs were magnetically pulled and stabilized across the cell membrane to achieve electrical contact with the extracellular conductive cell plating substrate (Fig. 1*B*).

The following describes the step-by-step procedure for NanoTouch recording method (Fig. 1*C*). CHO or HEK cells were plated on a CG slide, and GMNPs were delivered to the cytosol using PEI (dendrimers). Activated dendrimer (SuperFect; Qiagen) or pore-forming peptides (SLO) can be used in place of PEI (Lévy et al. 2010). Prior to this step, it is important to confirm that the plated cells have formed a sheet, because any gaps in the cell sheet will cause shunt circuits between the bath medium and the CG surface. Next, a magnet was placed below the CG recording chamber, pulling GMNPs toward the bottom of the cells adhered to the CG. Finally, as GMNPs penetrated and spanned the membrane, the electrical connection between the intracellular compartment of the cell and the CG substrate was monitored with a conventional patch-clamp amplifier. With the reference electrode set in the bath solution and the CG connected to the amplifier inputs, the GMNPs functioned as an intracellular electrode by bridging the cellular membrane and connecting the cytosolic compartment to the CG surface (Fig. 1*B*).

**Fig. 2.**
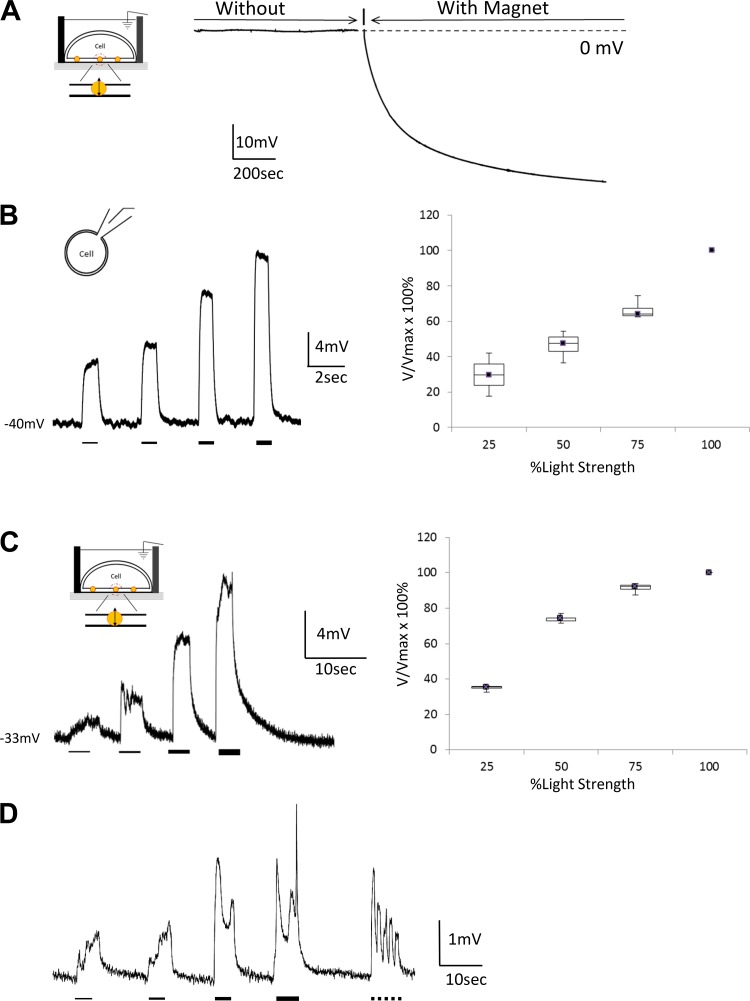
Detection of the resting membrane potential and of light-induced channelrhodopsin (ChRWR) depolarization using the NanoTouch recording method. *A*: detection of the resting membrane potential. Recordings before and after the magnet was placed under the conductive glass slide (CG) are shown. Placing the magnet under the CG triggered very slow downward movement. Without the magnet, this did not occur, indicating that for the gold-coated magnetic nanoparticles (GMNPs) to become transmembrane, a magnetic pulling force is required and it does not occur spontaneously. *B*: ChRWR responsiveness to blue light stimuli was validated using the patch-clamp technique. Under current-clamp mode, membrane potential was controlled at −40 mV. Light strength was increased from 0.2 to 0.8 A in 0.2-A intervals (25, 50, 75, 100% power), and the membrane voltage responses were recorded, which increased as stimulus strength increased (*left*). Normalized results (*V*/*V*_max_ × 100%) are shown in box and whisker plot (*right*), with the maximum response as 100% (*n* = 4). The maximum LED power used gave 7.4 ± 1.8 mV at −40-mV depolarization. *C*: NanoTouch recording of blue light activation of ChRWR. ChRWR-expressing CHO cells were subjected to blue light stimulation as in the patch-clamp method. Light strength was increased as in *B*, and the membrane voltage responses were recorded (*left*). Light strength-dependent ChRWR responses are shown in box and whisker plot (*right*), with the maximum response as 100%. The maximum LED power used gave depolarization of 5.1 ± 3.1 mV (*n* = 4; resting membrane potential was calculated to be −33 mV). This demonstrates that the GMNPs functioned similarly to a conventional intracellular microelectrode. *D*: another example of NanoTouch recording of blue light activation of ChRWR. Light strength was increased as in *B* and was followed by 5 repetitions of brief light pulses (750 ms on, 750 ms off).

Figure 2*A* shows the time course of an intracellular recording with NanoTouch method from a CHO cell expressing ChRWR. Under current clamp, baseline transmembrane voltages started to fall between 130 to 300 s (234 ± 40.4 s, *n* = 4) to reach a steady state, after introduction of a magnetic field. The NanoTouch method measured the CHO resting membrane potential as −33.3 ± 2.3 mV (*n* = 4).

Next, we performed experiments to test whether GMNPs could detect dynamic changes in membrane potential. Blue light-induced depolarization by ChRWR (a desensitization-reduced form of ChR; Wang et al. 2009) was chosen to demonstrate the validity of nanoparticle-based intracellular recording, because light-induced responses are relatively slow and easy to reproduce. With the use of the patch-clamp technique, ChRWR responsiveness to blue light stimulation was validated (Fig. 2*B*). Light pulses were applied to CHO cells that exhibited clear resting membrane potentials under current clamp, as shown in Fig. 2*A*. As measured by conventional patch-clamp electrodes in whole cell recording mode (Fig. 2*B*), as light intensity increased, the amplitude of membrane depolarizations [maximum depolarization of 7.4 ± 1.8 mV (*n* = 4) from a resting potential of −40 mV] caused by ChRWR activation increased proportionally.

With the use of the NanoTouch recording method, a similar proportional series of depolarizations was measured using a similar light pulse protocol (Fig. 2*C*). As observed in the patch-clamp experiments, ChRWR responses were detected with light pulses and were intensity dependent. Depolarizations were quantified as a function of light intensity, normalized to maximum response (*V*/*V*_max_ × 100%). The maximum LED power used gave a depolarization of 5.1 ± 1.6 mV (*n* = 4; Fig. 2*C*). This depolarization was within a similar range to that observed in the patch-clamp recordings (see above; *t* test: *P* > 0.05). Similar experiments were repeated 12 times using the NanoTouch method and produced varying degrees of amplitude response, ranging from 2 to 10 mV. From 9 of 12 experiments, we were able to detect a ChRWR response larger than 2 mV. These results were consistent with our hypothesis that GMNPs successfully detect membrane depolarization induced by light stimulation (Fig. 2*C*), supporting the idea that transmembrane GMNPs functioned similarly to conventional intracellular patch pipettes. When successful, the recordings lasted longer than 1 h, indicating that GMNPs incorporated in the cell membrane are noninvasive and do not significantly alter cell membrane integrity or health.

Activation and deactivation speeds were much slower in NanoTouch recording from a population of cells compared with manual patch-clamp recording from a single cell. It is likely that the charging and discharging speed of a larger total membrane area (cell sheet) would take, proportionally, much longer than that of a single cell (see below for detailed comparison).

**Fig. 3.**
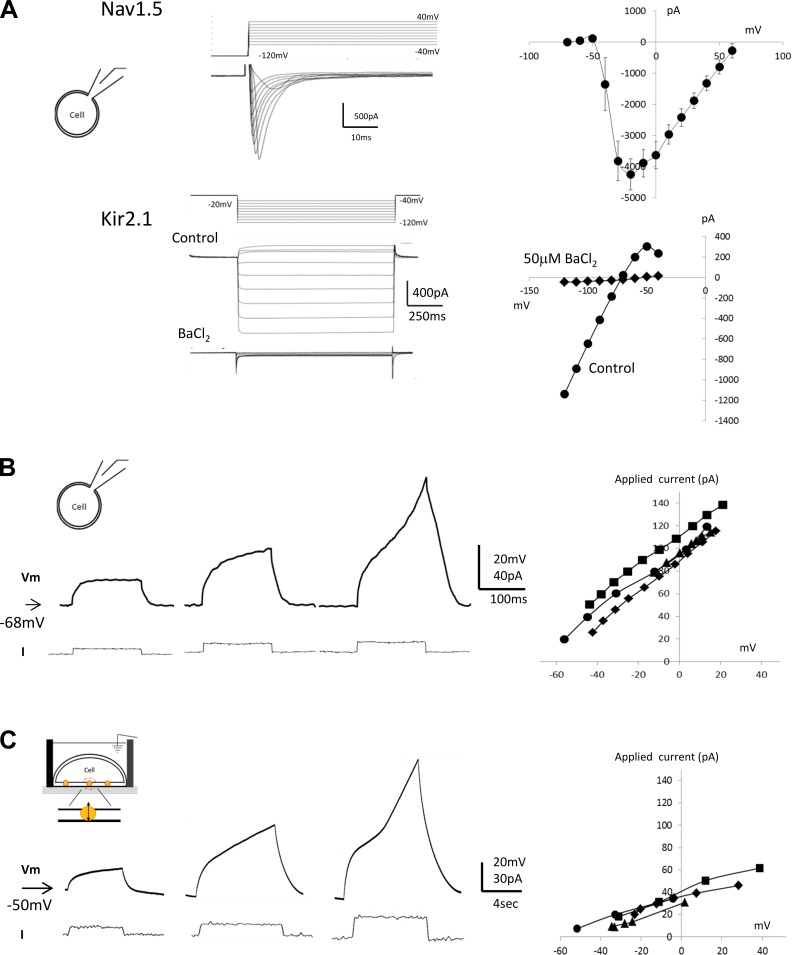
Validation of Nav1.5/Kir2.1 human embryonic kidney (HEK) cells under voltage-clamp and recording comparisons of patch-clamp and NanoTouch methods. *A*: depolarization voltage pulses resulted in transient inward current typical of the Nav1.5 current (*top left*), and the current-voltage (*I-V*) relationship [4,250.6 ± 499 pA (*n* = 4) at −20 mV] was plotted (*top right*). Hyperpolarizing voltage pulses resulted in inward rectifying current typical of the Kir2.1 current. It was completely blocked by barium (50 μM), an inward rectifier blocker (*bottom* *left*). The *I-V* plot was generated and had an amplitude of 760 ± 13.8 pA (*n* = 4) at −120 mV (*bottom right*). *B*: passive and active membrane responses using the patch-clamp. A series of depolarizing current pulses was applied and resulted in electrotonic depolarizing responses (*left*). As the injection current (*I*) amplitude was raised, a regenerative component appeared (*left*, 3rd trace). The *I-V* relationship was plotted (*right*), and passive membrane resistance (PMR) was 719.6 ± 1.7 MΩ (*n* = 4). Cell capacitance was also calculated by fitting the raw voltage traces. Single-cell capacitance values were 7.0 ± 1.8 pF (*n* = 5). *C*: passive and active membrane responses using NanoTouch. Passive membrane properties of the HEK cell sheet were examined using the NanoTouch method under current clamp. In response to a series of depolarizing current injections, passive membrane depolarizations were observed. Their voltage deflections increased as the applied current increased. In some cases, the voltage response took the regenerative form (*left*). The *I-V* relationship (*n* = 4) (*right*) gave a PMR of 1.34 ± 0.19 GΩ and total capacitance of 2.27 ± 0.23 μF (*n* = 3). *V*_m_, resting membrane potential (arrows).

To validate this method further, we tested whether the NanoTouch method could record much faster voltage-dependent ion channel responses, such as sodium-dependent action potentials. For these experiments, a HEK-293 cell line that stably expressed Nav1.5 and Kir2.1 channels (Nav1.5/Kir2.1 cells) was developed, and its electrophysiological properties were validated by conventional patch-clamp recordings, under voltage clamp (Fig. 3*A*). Peak amplitudes of inward sodium current were 4,250.6 ± 499 pA (*n* = 4) at −20 mV; peak inward rectifier currents were 760 ± 13.8 pA (*n* = 4) at −120 mV. We next characterized these cells in current-clamp mode. A series of depolarizing current pulses resulted in electrotonic depolarizing responses (Fig. 3*B*, *left*). As the amplitude of the injection current was increased, a regenerative component appeared (3rd trace in Fig. 3*B*, *left*). The current-voltage (*I-V*) relationship for passive membrane responses was plotted (*n* = 4; Fig. 3*B*, *right*). Slope membrane resistance was 719.6 ± 1.7 MΩ (*n* = 4), and single-cell capacitance was 7.0 ± 1.8 pF (*n* = 5).

With the use of the NanoTouch method, passive membrane properties of the HEK-293 cell sheet were examined under current clamp (Fig. 3*C*). In response to a series of depolarizing current injections, passive membrane depolarizations were observed. Voltage depolarizations increased as the applied current amplitude increased. In some cases, voltage responses took a regenerative form (Fig. 3*C*, *left*). The *I-V* relationship (*n* = 4) yielded membrane input resistance (*R*_m_) of 1.09 ± 0.16 GΩ and capacitance (*C*_m_) of 2.11 ± 0.21 μF (*n* = 3; Fig. 3*C*, *right*). We were able to record intracellular passive voltage responses in 10 of 16 attempts. From the double-exponential curve fitting, *R*_m_ was found to be 81.7 ± 7.9% (*n* = 4) of total resistance, and *C*_m_ was 93.3 ± 3.5% of total capacitance.

Our single-cell capacitance measurement (patch clamp under current-clamp mode) was 7 pF; therefore, the NanoTouch capacitance value divided by the single-cell capacitance indicted that >302 cells participated in the intracellular recording. Between 1,000 and 2,000 cells were plated on the CG (recording area of 3.14 mm^2^); therefore, 15–30% of cells were assumed to have incorporated GMNPs and to have contributed to the detection of intracellular voltage signals.

**Fig. 4.**
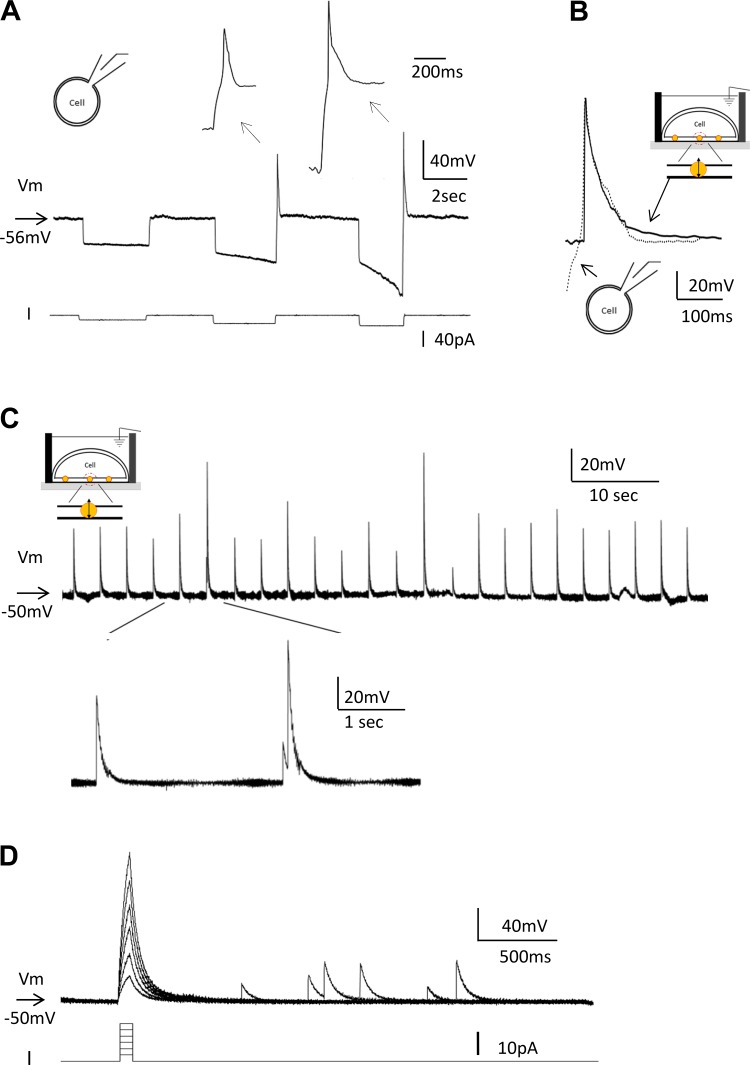
Recording Nav1.5/Kir2.1 human embryonic kidney (HEK) cell action potentials using manual patch-clamp and NanoTouch methods. *A*: all-or-nothing-type action potentials, anodal break action potentials, were generated in Nav1.5/Kir2.1 cells under current clamp. As prepulse-induced hyperpolarization became larger, the subsequent anodal break action potential amplitudes also increased, indicating that Nav1.5 inactivation was removed by the hyperpolarizing prepulse. Voltage (mV) indicates the prepulse membrane potentials. The arrows indicate the expanded views of action potentials in the middle trace. *B*: action potentials recorded using patch-clamp (broken line) and NanoTouch (solid line) methods were superimposed by adjusting their amplitude and indicate almost identical forms. *C*: NanoTouch captures spontaneous action potentials. The spontaneous generation of action potentials lasted 30 min. Action potential amplitudes were 39 ± 1.5 mV (*n* = 85 action potentials analyzed), and the arrow indicates *V*_m_ (*top* trace). *Bottom* trace shows an expanded view of the action potentials. *D*: NanoTouch recordings shown in *C* were further subjected to current stimulation. In response to the current injection (*I*), the membrane depolarized much quicker than the recordings shown in Fig. 3*C*, but they failed to trigger action potentials (spontaneous action potentials appeared irrespective of current injection).

As shown above, Nav1.5/Kir2.1 cells responded to depolarizing stimuli. However, depolarizing stimuli applied from the resting membrane potential resulted in slow, graded regenerative responses with use of patch-clamp recording; all-or-nothing action potentials were rarely observed, probably because of persistent inactivation of Nav1.5 at the relatively depolarized resting membrane potential of this cell line. Therefore, hyperpolarizing prepulses were applied to remove Nav1.5 channel inactivation. This caused all-or-nothing action potentials resulting from anode break (Fig. 4*A*). As the amplitude of hyperpolarizing prepulses was increased, resulting action potential amplitudes saturated to a maximal size, indicating that the degree of Nav1.5 inactivation removal determined action potential amplitude.

The NanoTouch method was used to characterize this Nav1.5/Kir2.1 HEK cell line. It is very important to have optimum plating conditions, because if cells are plated sparsely and areas of the conductive glass are exposed to the bath medium, a shunt circuit will form with the bath ground electrode, which results in failure to detect an intracellular membrane potential. This requirement is reminiscent of the Ussing chamber recording, invented by Hans Ussing to measure the permeability of epithelial cells, in which complete cell sheet formation is a prerequisite for successful recordings (Ussing and Zerahn 1951).

Compared with CHO cells, HEK-293 cells adhered to the CG surface much more weakly, which made NanoTouch recoding from HEK-293 cells much more challenging. Our attempt to induce anodal break action potentials by applying a hyperpolarizing prepulse did not result in the generation of action potentials. However, to our surprise, we successfully recorded spontaneous all-or-nothing-type action potentials with the NanoTouch method (*n* = 2; Fig. 4*B*), lasting 30 min in one preparation and 100 min in another. Analyses of these recordings gave action potential amplitudes of 39 ± 1.5 and 44.2 ± 2.9 mV from the two preparations. Resting membrane potentials were −50.5 and −50.3 mV.

In addition to the method described in Fig. 2*A*, by taking advantage of the conductive nanoparticles being held in the membrane by the magnet, resting membrane potential can be determined by comparing recordings before and after the conductive nanoparticles are released from the membrane by removing the magnet from the CG detection area. Action potentials recorded using manual patch-clamp and NanoTouch methods were superimposed and found to produce similar amplitudes and decay kinetics (Fig. 4*B*). Depolarizing currents were injected to examine whether action potentials could be triggered in this preparation (Fig. 4*D*). The current injections (Fig. 4*D*) resulted in large membrane depolarizations, which were much larger than the size of the following spontaneous action potentials, but failed to trigger action potentials. Since a large *R*_acc_ value does not prevent the voltage measurement (Brette and Destexhe 2012), it is most likely that the transmembrane conductive nanoparticle *R*_acc_ was larger than *R*_m_, preventing effective stimulation; the applied current thus could not effectively depolarize/stimulate the cell membranes. The decay phase was examined by fitting with a double-exponential equation to find out the ratio of *R*_acc_ and *R*_m_. Different from the experiments where current injection was successful, the ratios of the *R*_acc_ and *R*_m_ components were 56% and 44%, respectively, suggesting that, indeed, large *R*_acc_ value precluded effective current stimulation.

Because the approach that has been shown so far requires cell sheet formation for successful intracellular recordings, it cannot be used to record from isolated sparsely growing cells, especially from neurons. The NanoTouch principle is that the transmembrane conductive nanoparticle (GMNP) detects voltage signals by bridging the cytosolic and the extracellular components across the cell membrane. We considered that if the NanoTouch principle is correct, a conductive material (i.e., conductive glass) does not have to be placed only under the cells. A conductive material could be placed anywhere, such as above the cells, so long as the GMNPs can be magnetically pulled toward the conductive material. To test this hypothesis, we utilized the conductive property of a nickel-coated neodymium magnet as an electrode, similarly to the way CG was used as a culturing substrate for NanoTouch. We felt that this approach would allow application of the NanoTouch method to cultured neurons by isolating the area beneath the magnet from the bath solution. The magnet was directly connected to a recording amplifier, and its surface was covered with Parafilm, except for the bottom, to insulate itself from the bath solution. As long as the above condition is met, magnet electrode (MagEle) recording can be applied to record intracellular potential from a single cell or cells in suspension (i.e., acutely dissociated neurons). If dishes are placed on an iron metal plate, this MagEle not only functions as a recording electrode but also helps to hold itself steady on the culture plate by pressing itself toward the cells. Such a procedure does not require a skilled operator to carefully micromanipulate the magnetic probe onto the surface of the cell; the probe would only have to be placed into a well of cells.

**Fig. 5.**
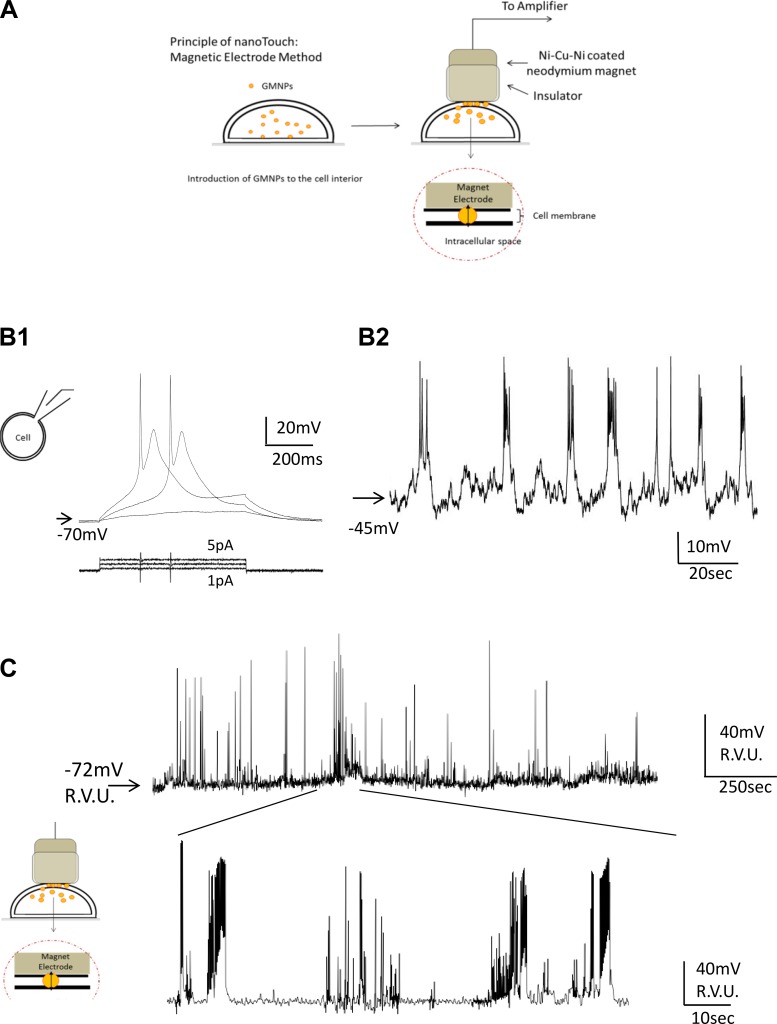
Schematic illustration of the conductive nanoparticle-magnet electrode (MagEle) recording method and application of MagEle recording to differentiated NG-108-15 hybridoma cells. *A*: the MagEle mode of the NanoTouch method utilizes a conductive nickel-coated neodymium magnet as an electrode (a conductive material equivalent to the conductive glass used in NanoTouch). Gold-coated magnetic nanoparticles (GMNPs) were first introduced to target cells using polyetherimide, and then the magnet was pressed directly against cells to pull GMNPs upward. Once the nickel-coated neodymium magnet made contact with the GMNPs, membrane potential changes were detected. Therefore, the magnet itself functions as a connecting material to the amplifier input. *B*: characterization of differentiated NG108-15 cells using the patch-clamp method under current clamp. Differentiated NG108-15 cells responded to depolarizing current injection and generated action potentials (*B1*). NG108-15 cells generate almost no spontaneous activity; therefore, 20 μM veratridine (a sodium channel opener) was used to activate NG108-15 cells. On application of veratridine, spontaneous action potentials appeared (*B2*). *C*: MagEle recording of veratridine-activated differentiated NG108-15 cell action potentials. Veratridine (20 μM) was used to activate NG108-15 cells to induce spontaneous action potentials. The region indicated was expanded to show the shapes of NG108-15 cell action potentials. Veratridine not only induced spontaneous action potentials but also triggered bursting activities that were not seen in electrically induced action potentials in the patch-clamp study, and they lasted for 90 min. Relative voltage units (R.V.U.) were used to show action potential amplitude.

By placing the magnet on top of the cells as described, the GMNPs in the cells were pulled toward the MagEle. Once the GMNPs became transmembrane and formed contact, an intracellular recording configuration was achieved. Caution is required regarding the strength of the magnet; the magnet needs to be strong enough to pull and hold GMNPs tightly, but if the magnet is too strong, cells will be destroyed, so optimization is necessary. In the experiment discussed below, a neodymium magnet of 200 mT (6-mm diameter) was used as a MagEle. A schematic depiction of a MagEle recording electrode is presented in Fig. 5.

For MagEle experiments, we used differentiated NG108-15 (mouse neuroblast × rat glioma hybrid) cells to record native action potentials generated by cells of neuronal origin. First, using conventional whole cell patch-clamp recordings, we confirmed that differentiated NG108-15 cells were capable of generating action potentials in response to applied depolarizing current injections (Fig. 5*B1*). Differentiated NG108-15 cells did not generate spontaneous action potentials; therefore, veratridine (20 μM), which reduces sodium channel inactivation, was applied to induce spontaneous action potential generation (Fig. 5*B2*). The MagEle mode of intracellular recording did not permit effective current injection. Therefore, spontaneous action potentials were induced by treating the cells with veratridine. Figure 5*B2* shows examples of MagEle recordings from differentiated NG108-15 cells displaying a spontaneous series of burst activity on application of veratridine (20 μM). This spontaneous activity persisted during the course of the experiment (90 min). As shown in the expanded (*bottom*) trace in Fig. 5*C*, veratridine delayed action potential repolarization and produced bursting activities similar to those seen with the conventional patch-clamp recording (Fig. 5*B2*).

As described in Figs. 2 and 3, input resistances varied from recording to recording, which affected the size of action potentials. All NanoTouch recordings, including those in the MagEle recording mode, were simultaneously acquired from multiple cells, where it is presumed the number of GMNPs per cell varies. This likely is reflected in the varying sizes of action potentials observed with MagEle recordings

## DISCUSSION

Various events take place in excitable cells that include not only action potentials but also subthreshold synaptic potentials and membrane oscillations. These changes in membrane potential are, at present, detectable only by intracellular recording, which is mainly achieved by sharp electrodes or patch pipettes.

An alternative, frequently used method is the extracellular recording method, where electrodes contact the outside of a cell. Because extracellular signals are differentials of membrane potential changes, and small-amplitude events are masked by the baseline noise, slow membrane potential changes (synaptic potentials, slow decaying potentials seen in ventricular action potentials) and the resting membrane potential are not detected/determined under this method. The multielectrode array (MEA) recording system is based on this extracellular recording method (Khudhair et al. 2017).

MEA does not require the complicated procedures needed for the manual patch-clamp technique; therefore, intense efforts have been made to merge the advantages of extracellular MEAs and intracellular microelectrodes. The inventions of mushroom shaped electrode and pillar electrode methods have been adapted to the MEA system (Spira and Hai 2013; Xie et al. 2012). These have made it possible to record intracellular signals in MEA systems. In these systems, the voltage-sensing parts protrude, and cells are grown on top of these protrusions. Once protrusions are engulfed by target cells, the cell membranes are broken by using electroporation to rupture the membranes that engulf these electrodes. However, all these methods have limitations regarding the duration of stable recordings.

Another notable new method is based on field effect transistor **(**FET) technology, which replaces glass electrodes with FET electrodes (pillar electrode). Of the methods mentioned above, this technique is the most stable and allows long-term recordings (Zhang and Lieber 2016). The FET electrode itself is a voltage sensor, and it causes much less damage to cell membrane integrity compared with the other MEA-based techniques.

Similarly to FET electrodes, the transmembrane GMNPs used in the NanoTouch method cause no detectable damage to membrane integrity; continuous intracellular recording of longer than an hour can be achieved. In addition, GMNPs are stable within cells for several days (at least 4 days in dividing cells; e.g., HEK-293 cells) if experiments are conducted under sterile conditions, allowing the same preparation to be reexamined at multiple time points (data not shown).

Under the NanoTouch method, the resting membrane potential can be reliably measured. The resting membrane potential develops slowly with the NanoTouch technique, which is different from patch-clamp and sharp electrode intracellular recordings, in which resting membrane potentials are detected abruptly by breaking the cell membrane or by inserting a sharp electrode into the cell. This slow time course can confuse the accurate determination of the resting membrane potential. We think that the slow time course reflects the number of GMNPs in the membrane becoming available to form electrical contact with the CG substrate. Also, it can be measured as *R*_acc_. When this slow-developing membrane shift was small or not clear, the recorded action potential amplitudes were small (~5 mV; data not shown), indicating that *R*_m_ depends on the number of transmembrane nanoparticles. The *R*_m_ value measured by NanoTouch was somewhat higher than that of the conventional patch-clamp method, although the difference was not significantly different (*t* test: *P* > 0.05; Fig. 3, *B* and *C*, *right*). A possible explanation for this is that, as mentioned above, the number of GMNPs per cells is a dominant factor in determining input resistance.

Further investigations are required to understand the uptake and movement of GMNPs in a cell. Under the NanoTouch method (Figs. 2*C* and 3*C*), the charging and discharging time courses are slower than those obtained under the patch-clamp method (Figs. 2*B* and 3*B*). This potentially affects the time course of subthreshold responses, which do not recruit voltage-dependent K^+^ channels for active membrane repolarization. However, the decay time course of smaller responses (<2 mV; Fig. 2*D*) was not detectably delayed compared with the larger responses (~10 mV; Fig. 2*C*). It is possible that unsynchronized, small slow depolarizations or depolarizations originating from a subset of cells in a cell sheet could be submerged in the large capacitance of a cell sheet. In that case, the detections of small responses may be difficult; however, it should not be a serious obstacle as long as the cell sheet consists of a uniform population of cells.

Another important property of NanoTouch is that it functions similarly to perforated patch recordings. The perforated patch clamp keeps the intracellular milieu intact, except for the exchange of monovalent cations. The NanoTouch method relies on the transmembrane conductive nanoparticles to detect membrane potentials; therefore, the intracellular environment is not altered.

Under the MagEle mode (Fig. 5*C*), relative voltage units were used. The GMNP-nickel-coated neodymium magnet contact resulted in >100-mV offset potential, which was different from that measured by the GMNP-FTO conductive glass contact. We were unable to determine the absolute voltage of the membrane potential. Future modifications of the magnet surface are necessary.

We have demonstrated that the intracellular membrane potential can be recorded using membrane-spanning conductive nanoparticles, without the use of glass (patch clamp) microelectrodes. Application of nanoparticles for membrane potential detection has thus far been limited to optical measurements (Marshall and Schnitzer 2013; Park et al. 2018). We expanded the application of nanoparticles as an electrophysiological recording tool, comparable to current-clamp recording modes. The NanoTouch method is an intracellular recording method; therefore, it has the capability to record slow and small voltage changes (ligand-gated ion channel responses). This is not possible with the MEA system based on extracellular recording configurations. Also, the NanoTouch method has the potential to be an alternative to membrane potential fluorescent dye assays, because *1*) it detects electrical signals (its time resolution is not limited by the frequency-response characteristics of the fluorescence sensors and detection device), and *2*) dye photocytotoxicity is absent (Pakhomov et al. 2017; Waggoner 1979).

One of the main technical challenges to achieve successful NanoTouch recordings is that the cells must grow to confluence to form cell sheet. Since the large total capacitance from a population of cells (cell sheet) slows the charging and discharging process, the speed of voltage responses could be significantly affected by this. One of the key factors that needs to be improved in the future is to reduce cell membrane capacitance by controlling the cell number, which has a significant impact on recording quality (i.e., limiting the size of conductive glass area for cell plating, or preparing patterned conductive materials to single-cell size).

This report also presents an alternative way (the MagEle) to achieve intracellular recordings, which allows recordings from sparsely plated cells such as neurons (Fig. 5*C*). Successful MagEle recordings strongly depend on how well the bottom of the magnet is isolated from the surrounding bath solution. Similar to the cell sheet requirement, when this isolation fails, action potentials will not be recorded. As a variation of the MagEle method, it will be interesting to combine multiple MagEles to perform intracellular recordings from different locations in the same culture. Although we have made such attempts, the magnets used in this work were too strong and repelled each other when placed in close proximity. Further optimization is necessary to achieve such multiple-MagEle recordings.

The main caveat of this method is that the recording is made from multiple cells; therefore, the behavior of single cells cannot be independently examined. This limitation is even true with MagEle mode, under which neuronal action potentials were recorded (Fig. 5*C*). The principle of the NanoTouch method will be a powerful addition to neurophysiological observations if the ability to independently resolve activities of subsets of cells and networks of neurons is warranted. To fulfill such a requirement, it is necessary to improve/modify the cell plating electrodes and introduce a patterned conductive electrode.

We feel that the most attractive future application of the NanoTouch method is to combine it with MEA (NanoTouch-MEA hybrid) using high-density magnetic/conductive substrates, which will provide an intracellular recording array underneath the culture to address large numbers of individual cells. Specifically, having multiple small electrodes (or sensors) placed on the bottom of probes will be a much simpler solution to free us from the one major obligatory condition of forming a cell sheet. It would be much easier to cover these electrode surfaces with a group of cells to completely isolate them from the bath solution.

In this article, I have reported a proof-of-concept demonstration of how the intracellular potentials can be recorded using transmembrane conductive nanoparticles. Because this technology is still in its early stages of development, it requires further optimization before it can be a routine tool for physiological research. However, as stated above, the NanoTouch method can be a strong asset when combined with previously established methods, especially with the MEA technology. For the transmembrane conductive nanoparticle to function as an intracellular electrode, it needs only to be plated on a surface that has conductive property (i.e., gold, carbon, etc.). We hope that further development of the NanoTouch method will contribute to the advancement of cellular electrophysiological research.

## DISCLOSURES

This work was submitted under Patent application WO/2018/199334, “Intracellular recording method using the membrane penetrating nanoparticles as an electrode (nanoelectrode).”

## AUTHOR CONTRIBUTIONS

M.L.S. conceived and designed research; performed experiments; analyzed data; interpreted results of experiments; prepared figures; drafted manuscript; edited and revised manuscript; and approved final version of manuscript.
